# Exploring the Relationships Between Determinants of Musculoskeletal Injuries in Combat Sports: Focus on Sambo and Judo

**DOI:** 10.1155/tsm2/8209351

**Published:** 2025-08-06

**Authors:** Anastasiia Lapaeva, Vadim Belyaev, Viktoriia Goryachkina, Olga Lobanova, Valery Rokotyansky

**Affiliations:** Department of Sports Center, Sechenov First Moscow State Medical University of the Ministry of Health of the Russia Federation, Moscow, Russia

**Keywords:** athletic injuries, back injuries, judo, sambo

## Abstract

The aim of the study was to identify the relationship between factors contributing to injuries among athletes in sambo and judo. We examined the influence and interaction of such injury factors in sambo and judo as rapid weight loss, competitions, training processes, the lateralization of an athlete's fighting stance, and the X-factor. The survey involved 61 athletes (42 men and 19 women) over the age of 18, including 74% sambo practitioners and 26% judo practitioners of high athletic qualification with injuries to the upper and lower extremities. The odds of sustaining an injury during competitions among athletes who resorted to rapid weight correction were 5.59 times higher than among athletes who did not use RWL (OR: 5.59; 95% CI: 1.77–17.71, *p*=0.004). The odds of injuring the ipsilateral limb with a pronounced lateral right-sided or left-sided stance were 18.13 times higher than for the contralateral limb (OR: 18.13; 95% CI: 4.81–68.36, *p* < 0.001). The results of our study show that the relationship between factors such as rapid weight loss and participation in competitions (*p*=0.004), as well as an asymmetric fighting stance reflecting lateral preference and the side of the injured limb (*p* < 0.001), statistically significantly increases the risk of injuries in sambo and judo. The presence of signature techniques involving rotational throws in an athlete's arsenal did not significantly affect lower back pain (LBP) or injuries.

## 1. Introduction

Fighting sports are among the most popular sports in the world. According to the International Sambo Federation (FIAS), the number of countries where sambo is practiced is 120, and FIAS has 99 national federations as members [1]. Judo is a popular Olympic and Paralympic sport, with over 20 million active participants from more than 200 countries worldwide [2]. However, as the number of participants increases, so does the level of injuries. In recent years, significant efforts have been made to reduce the injury rate in combat sports. However, the question remains to what extent these measures are effective [3]. Technical actions in sambo and judo primarily include grips, standing throws, as well as joint locks, chokeholds, and pins in ground positions. The main factors contributing to injuries in combat sports are generally considered to be the level of preparedness, gender, weight categories, rapid weight loss (RWL), and noncompliance with competition rules [4]. It has previously been suggested that injuries result from complex and nonlinear interactions of multiple factors [5–7]. In the model proposed by Wroblewska and colleagues [8], seven of the 24 factors were identified as the most significant: previous injuries, age, sleep duration, blood composition, competitions, training load, and atmospheric conditions, all of which influence the risk of future injuries [9–11]. However, it is likely that, at an individual level, a single isolated factor may be sufficient to cause or predict injuries [5]. Anterior cruciate ligament (ACL) rupture is one of the most severe injuries in competitive judo, associated with a long recovery period and a high risk of being unable to return to the previous level of performance [5, 12–15]. In addition, intervertebral disc prolapses, lower back pain (LBP), and muscle spasms have been noted to negatively impact athletic performance in judo, requiring significant recovery time [16, 17]. Moreover, a key risk factor for injuries during rotational movements in sports is asymmetric repetitive axial twisting in extreme ranges of motion, known as the X-factor [18]. An increased X-factor leads to excessive spinal load and may be associated with asymmetric patterns of degenerative spinal changes. In sambo and judo, the X-factor is most pronounced during rotational throws (such as forward foot sweeps, inner and outer reaps, hip throws, shoulder throws, and kneeling shoulder throws). Given the need to study the relationships between potential injury factors, such as lateral preference, RWL, technical actions, and the injuries sustained by athletes, a survey was conducted among athletes with a history of limb injuries or injuries requiring surgical intervention. The aim of the study was to identify the relationship between factors contributing to injuries among athletes in sambo and judo.

## 2. Materials and Methods

The following methods were applied in the study: systematic search and analysis of scientific and methodological literature, surveys, and methods of mathematical statistics.

The literature search was conducted in the PubMed, Cochrane Library, and Google Scholar databases using keywords and phrases such as “sambo,” “(sambo) AND (injuries),” “(combat sports) AND (injuries),” “((((injury factors) OR (predictors of injury)) AND (judo)) OR (sambo)) OR (combat sports),” and “sambo OR judo ‘injury factors'.” No time restrictions were set for the search.

The questionnaire was anonymous and included 23 questions about potential causes of injuries, the nature of the injuries, lateral preference, and the primary technical actions of the athletes. This study did not account for differences based on gender, age, or weight category of the athletes. The survey was conducted among athletes with experience in sambo or judo who had a history of injuries or damage to the upper and lower extremities requiring surgical intervention, as well as those with spinal pain or degenerative changes. The survey was conducted at the Department of Theory and Methodology of Combat Sports at the Russian University of Sport “GCOLIFK” from January 9, 2023, to June 19, 2024. Tools from the global Internet network and the free Google Docs web package were used. We received 65 completed questionnaires, of which 4 were filled out incorrectly or were incomplete and were consequently excluded from further analysis. The survey involved 61 athletes (42 men and 19 women) over the age of 18, including 74% sambo practitioners and 26% judo practitioners of high athletic qualification with injuries to the upper extremities (shoulder joint: 31.1% and elbow joint: 26.2%) and lower extremities (hip joint: 1.6%, knee joint: 39.3%, and ankle joint: 4.9%). This study did not take into account differences in gender, age, and weight category.

### 2.1. Statistical Analysis

Statistical analysis was performed using IBM SPSS Statistics Base 26.0 (SPSS Inc, Chicago, IL, USA). Categorical data were described using absolute values and percentages. Pearson's chi-square test was used to analyze fourfold and multifold contingency tables. The strength of the association between variables (effect size) was assessed using the interpretation of Cramer's V values according to the recommendations of Rea and Parker. To evaluate the relationship between the studied factors and observed outcomes, odds ratios (ORs) and 95% confidence intervals (95% CIs) were calculated. Differences were considered statistically significant at *p* values < 0.05.

## 3. Results

All athletes reported at least one injury in their medical history. Among the respondents were both active athletes (57%) and those who had already ended their competitive careers (43%). All athletes reported having undergone either surgical (44%) or conservative treatment (56%). The most frequently affected segment was the knee joint (39.3%). Surgical intervention was primarily required due to ACL tears (24.5%) and meniscus injuries (21.3%) (Table 1). The majority of respondents (82%) reported having resorted to RWL in preparation for competitions, but only 36% reported RWL at the time of injury. This is likely related to the fact that most injuries occurred during the training process (65.5%). All athletes predominantly used an asymmetric fighting stance: 65.5% in a right-sided stance and 34.5% in a left-sided stance.

**Figure 1:**
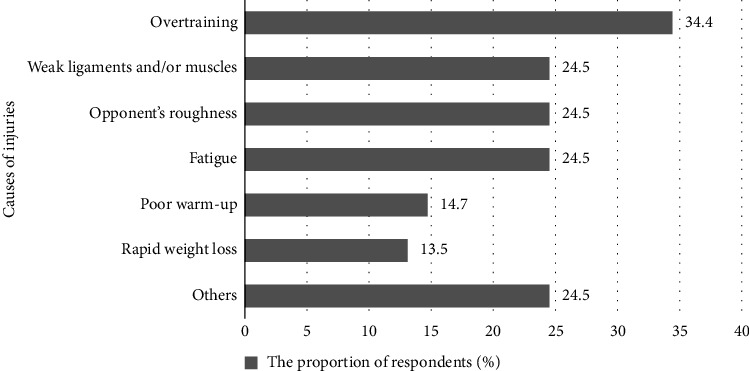
Possible causes of injuries in the surveyed athletes with injuries to the upper and lower extremities.

Injuries to the limbs primarily occurred in the standing position during the athletes' own attacks (22.9%), while defending against an opponent's attack (42.6%), or during an opponent's counterattack (14.7%). During groundwork (parterre), 19.6% of athletes sustained injuries (Table 1). According to the respondents, the main causes of injuries were overtraining (34.4%), weak ligaments and/or muscles (24.5%), opponent's roughness (24.5%), fatigue (24.5%), poor warm-up (14.7%), and RWL (13.1%) (Figure 1). Athletes also noted other reasons (24.5%), such as their own mistakes during an attack or defense, the presence of microinjuries, unfortunate circumstances, refusing to submit during an opponent's submission hold, anatomical features of ligament placement, prohibited techniques by the opponent, incompetent refereeing, and inadequate material and technical support for training or competition processes. The overwhelming majority of respondents reported experiencing pain or pathologies (hernias, protrusions, and prolapses) in the lumbar (59%), thoracic (13.1%), cervical (21.3%), or sacral (4.9%) regions of the spine. In addition, 62.3% noted that they frequently experience pain or discomfort when performing throws such as shoulder throws (34.4%), chest throws (22.9%), leg grabs (16.4%), and “windmill” throws (9.8%).

When comparing the frequency of injuries to the right limb among athletes with a right-sided stance, statistically significant differences were found (*p* < 0.001). The odds of injuring the ipsilateral limb with a pronounced lateral right-sided or left-sided stance were 18.13 times higher than for the contralateral limb (OR: 18.13; 95% CI: 4.81–68.36, *p* < 0.001). A strong association was observed between the compared variables (*V* = 0.6) (Table 2). In the survey, 69% of athletes reported using rotational throws (such as forward foot sweeps, inner reaps, outer reaps, hip throws, shoulder throws, and kneeling shoulder throws) as part of their primary technical actions. A trend was also observed in the use of rotational throws as primary technical actions among athletes with spinal pain or pathology, but the differences were not statistically significant. When comparing the frequency of injuries during competitive activities involving RWL, statistically significant results were obtained (*p*=0.004). The odds of sustaining an injury during competitions among athletes who resorted to rapid weight correction were 5.59 times higher than among athletes who did not use RWL (OR: 5.59; 95% CI: 1.77–17.71, *p*=0.004). A moderate association was observed between the compared variables (*V* = 0.39) (Table 3).

## 4. Discussion

In the conducted study, the most frequently affected segments were the knee joint (39%) and the shoulder joint. The most common injury was damage to the ACL (24.5%). In addition, most injuries occurred in the standing position (80.2%) during the athlete's own attack, while defending against an opponent's attack, or during an opponent's counterattack. Similar data were obtained in the study by Blah and Smolders [5], where the highest percentage of injuries occurred during standing combat (78%), with 18.30% occurring during groundwork. In standing combat, injuries were primarily associated with actions during throws (25.85%).

In the study by Kim et al. [19], 914 injuries were recorded among 340 athletes. Most injuries occurred in the lower extremities (38.0%), followed by the upper extremities (25.9%), the torso (24.8%), and the head and neck area (11.3%). The severity of injuries was significantly influenced by gender, fighting style, and weight category. According to the study by Hunker et al. [20], in Brazilian jiu-jitsu, the most common injuries were to the fingers/hand (78.6%), shoulder (48.7%), and knee (61.5%); 15 out of 56 athletes (27%) underwent surgery due to BJJ injuries. Petrisor et al. [21] found that the need for surgical intervention on an injured limb led to a 6.5-fold increase in the risk of not returning to the sport. According to our survey, 74% of athletes had degenerative changes or pain in the spine. As a result of spinal injuries, athletes primarily sought help from massage therapists (40.9%), chiropractors (29.5%), and sports doctors (24.5%). This partially aligns with the data from E. Eindhoven et al. [22], who reported that chiropractors were involved in treating combat sports athletes from amateur to professional levels. It was also reported that 75%–85% of Canadian national team athletes visited a chiropractor. At the 2013 World Games, 22% of Canadian athletes across all sports and 18% of athletes from all participating countries sought help from chiropractors.

### 4.1. RWL

In combat sports, athletes are typically classified into weight categories for competitions to avoid significant weight differences between opponents and, consequently, reduce the potential risk of injuries [23]. Most sambo and judo athletes of various ages and skill levels resort to RWL methods before competitions to gain a strength advantage over weaker or lighter opponents. To achieve this, they lose 2%–10% of their body weight in the last 2-3 days before competitions [23, 24]. Common RWL methods include fluid restriction and dehydration, food restriction, low-carb diets and fasting, increased training intensity, training in plastic suits and using saunas, and the use of diuretics, laxatives, and vomiting [25–27]. The use of these methods can lead to a range of health issues, dehydration, increased risk of injuries and illnesses, and reduced performance [27, 28]. These findings are consistent with the results of our study, which confirmed that the odds of sustaining an injury or damage when using RWL immediately before competitions were 5.59 times higher (OR: 5.59; 95% CI: 1.77–17.71, *p*=0.004). According to Kim et al. [19], judo athletes had a significantly higher injury rate during periods of RWL compared to other training periods (23.18 vs. 11.93; *p* < 0.001). In addition, nearly 90% of sambo athletes reported resorting to RWL before competitions [25].

### 4.2. Asymmetry

An example of asymmetry in motor actions during a match is the fighter's stance. A right-sided or left-sided fighting stance is a constant throughout an athlete's career, reflecting individual features of the brain's lateral organization [29, 30]. Although some level of asymmetry is considered acceptable in athletes, values exceeding 15% between limbs may indicate a predisposition to injury [31] and can also affect performance [32]. In our study, it was found that the risk of injury to the right limb in a right-sided fighting stance, and accordingly, the left limb in a left-sided fighting stance, increases by 18.13 times (OR: 18.13; 95% CI: 4.81–68.36, *p* < 0.001). Some authors have suggested that this may be related to accumulated fatigue in the upper and lower limbs, which can cause muscle imbalance and exacerbate asymmetry between the lower limbs [32, 33]. Given that judo-specific tasks are typically performed unilaterally (particularly throws), it is possible that accumulated fatigue during repeated matches may manifest more noticeably on the dominant side [34].

### 4.3. X-Factor

The “X-factor” is the angle in the transverse plane between the line of the pelvic axis, drawn through the anterior superior iliac spine on the right and left sides, and the line of the shoulder girdle, drawn through the acromial processes [18]. It is believed that an increased X-factor leads to greater axial rotation of the torso and shoulder girdle, thereby accumulating elastic potential energy in the trunk muscles [18]. In sambo and judo, rotational throws also involve similar movement mechanics and may carry a potential risk of injury to the lumbar and thoracic spine [35]. The results of this study did not show a statistically significant increase in the frequency of LBP among athletes who use rotational throws as their “signature techniques” compared to those who use them less frequently. However, it has previously been shown that the X-factor can exacerbate existing pain and degenerative damage in the lower back among athletes in various sports [36].

## 5. Conclusions

This study examined injury factors such as RWL, participation in competitions, rotational throws (X-factor), and the lateralization (asymmetry) of an athlete's fighting stance. The results of our research show that the relationship between factors such as RWL and participation in competitions, as well as an asymmetric fighting stance reflecting lateral preference and the side of the injured limb, statistically significantly increases the risk of injuries in sambo and judo. The presence of rotational throws as “signature techniques” in an athlete's arsenal did not significantly affect LBP or injuries. However, a noticeable trend toward LBP injuries was observed when performing throws associated with an increased X-factor.

## 6. Perspective

This study is not without limitations, as we cannot determine whether the athletes were entirely truthful when completing the questionnaire. Nevertheless, this research provides an additional perspective and contributes to understanding the interaction of certain injury factors, laying the groundwork for developing effective strategies to prevent injuries and illnesses among athletes in the future.

## Figures and Tables

**Table 1:** Data on the location of injuries, circumstances of injuries, and treatment received by surveyed athletes with injuries to the upper and lower extremities.

*Anatomical region of injury (%)*	
Shoulder joint	31.1
Elbow joint	26.2
Hip joint	1.6
Knee joint	39.3
Ankle joint	4.9

*Injured limb (%)*	
Right	55.7
Left	34.5
Both	9.8

*Surgical treatment (%)*	
Yes	44
No	56

*Location at the time of injury (%)*	
Competition	34.5
Training	65.5

*Circumstances of injury (%)*	
Own attack in a standing position	22.9
Defense against opponent's attack in a standing position	42.6
Opponent's counterattack in the standing position	14.7
Groundwork (parterre)	19.6

**Table 2:** The relationship between fighting stance and injured limb in surveyed athletes with upper and lower limb injuries in sambo and judo.

Fighting stance	Injured limb				*p* value	ОR; 95% CI
	Right (*n* = 39)		Left (*n* = 22)			
	Abs.	%	Abs.	%		
Right-sided	34	85	6	15	< 0.001^∗^	18.13; 4.81–68.36
Left-sided	5	23.8	16	76.2		

^∗^The differences in the indicators are statistically significant (*p* < 0.05).

**Table 3:** The relationship between rapid weight loss and competitive activity in surveyed athletes with upper and lower limb injuries in sambo and judo.

Rapid weight loss (risk factor)	Getting injured				*p* value	OR; 95% CI
	During competitive activities		During training activities			
	Abs.	%	Abs.	%		
Presence of a risk factor	13	59.1	9	40.9	0.004^∗^	5.59; 1.77–17.71
Absence of risk factor	8	20.5	31	79.5		

^∗^The differences in the indicators are statistically significant (*p* < 0.05).

## Data Availability

The data that support the findings of this study are available from the corresponding author upon reasonable request.
